# Discovery of novel mycoviruses from fungi associated with mango leaf spots

**DOI:** 10.3389/fmicb.2025.1545534

**Published:** 2025-02-26

**Authors:** Qihua Wang, Mengyi Chen, Yanling Xie

**Affiliations:** ^1^Key Laboratory of Hainan Province for Postharvest Physiology and Technology of Tropical Horticultural Products, Zhanjiang Key Laboratory of Tropical Crop Genetic Improvement, Key Laboratory of Tropical Fruit Biology, Ministry of Agriculture and Rural Affairs of China, South Subtropical Crops Research Institute, Chinese Academy of Tropical Agricultural Sciences, Zhanjiang, Guangdong, China; ^2^Key Laboratory of Plant Pathology, College of Plant Science and Technology, Huazhong Agricultural University, Wuhan, China

**Keywords:** *Mangifera indica*, leaf spots, next-generation sequencing, mycovirus, viral diversity, viral evolution

## Abstract

Mango (*Mangifera indica*) is a commercially significant fruit crop cultivated globally. However, leaf spot diseases are common in mango orchards, which severely impact the yield. Mycoviruses hold promise as potential biocontrol agents. To investigate this possibility, fungi were isolated from mango leaf spot lesions, resulting in the identification of six strains that contained double-stranded RNA (dsRNA). Through BLASTx analysis of the NCBI non-redundant database, 27 mycovirus-related contigs were identified, which corresponded to 10 distinct viruses grouped into 8 lineages: *Alternaviridae, Chrysoviridae, Partitiviridae, Polymycoviridae, Orthototiviridae, Deltaflexiviridae, Narnaviridae,* and *Bunyaviricetes*. Full genomic sequences of these viruses were characterized and confirmed to be associated with their host fungi. The findings included six novel mycoviruses, three previously unreported viruses discovered in new hosts, and one virus strain. These results highlight the diversity and taxonomy of mycoviruses found in fungi associated with mango leaf spots.

## 1 Introduction

Mycoviruses infect fungi and are classified based on the host, genome structure, and phylogenetic relationships of viral proteins (Kondo et al., 2022). The majority of mycoviruses have double-stranded RNA (dsRNA) or positive-sense single-stranded RNA (+ssRNA) genomes; however, some mycoviruses with linear negative-sense single-stranded RNA (-ssRNA), single-stranded DNA (ssDNA), or circular single-stranded RNA genomes have also been identified (Yu et al., 2010; Liu et al., 2014; Schiwek et al., 2024). The recent Virus Metadata Resource (VMR, MSL39.v4) lists 40 viral families and 1 unclassified genus known to infect fungi^1^.

Advances in high-throughput next-generation sequencing (NGS) and bioinformatics have revolutionized the discovery of viruses across diverse organisms, including fungi. Large-scale meta-transcriptomic surveys, such as those conducted by Shi et al. (2016, 2018), have revealed thousands of novel viruses in invertebrates. Similarly, NGS-based viromics has identified 79 novel viruses in tomatoes and weeds (Rivarez et al., 2023). Studies on pathogenic fungi, endomycorrhizal fungi, and macrofungi have revealed the presence of new viral taxa, some of which possess unprecedented genomes (Sutela et al., 2020; Ye et al., 2023; Zhang et al., 2024; Zhou et al., 2024). Remarkably, even obligatory biotrophic oomycetes have been found to contain mycoviruses, further enriching the evolutionary narrative of *Riboviria* (Chiapello et al., 2020; Poimala and Vainio, 2024).

Fungal pathogens causing leaf spot diseases are an increasing threat to the vital tropical and subtropical mango fruit (*Mangifera indica*). The severity of mango leaf spots has risen globally in recent years (Guo et al., 2021). The following are some of the notable fungal pathogens: *Phomopsis mangiferae,* which causes stem-end rot (Ko et al., 2009); *Pestalotioid* fungi and *Fusarium* species, which cause leaf and gray leaf spots (Ko et al., 2007; Omar et al., 2018; Shu et al., 2020); and Botryosphaeriaceae fungi, which are associated with rachis necrosis, bark canker, and dieback (Javier-Alva et al., 2009; Shah et al., 2010; Serrato-Diaz et al., 2013). Although *Nigrospora oryzae* is a significant pathogen affecting crop production (Wang et al., 2021), its role in mango leaf diseases has not been documented to date. Recent studies have reported novel mycoviruses in *Phomopsis vexans* (Xie et al., 2022), *Neofusicoccum parvum* (family Botryosphaeriaceae) (Marais et al., 2021; Comont et al., 2024), *Pestalotiopsis* sp. (Chen et al., 2021), and *N. oryzae* (Liu et al., 2019; Yang et al., 2024).

Although the majority of mycoviruses establish latent infections, some significantly influence the growth, reproduction, and virulence of the host. For instance, *Cryphonectria hypovirus 1* (CHV1) hinders host growth and has been successfully used in controlling chestnut blight disease (Rigling and Prospero, 2018). *Sclerotinia sclerotiorum* hypovirulence-associated DNA virus 1 (SsHADV1), which has a ssDNA circular genome, effectively manages rapeseed sclerotinia disease (Yu et al., 2010; Yu et al., 2013) and improves the yields of rapeseed and wheat (Tian et al., 2020; Zhang et al., 2020). Similarly, *Fusarium graminearum gemytripvirus 1* (FgGMTV1) has been developed as a viral vector (Li et al., 2020; Zhang et al., 2023), and *Diaporthe sojae circular DNA virus 1* (DsCDV1) significantly attenuates fungal virulence (Wang et al., 2024). In addition, *Pestalotiopsis theae chrysovirus 1* (PtCV1) transforms its host fungus into a non-pathogenic endophyte (Zhou et al., 2021). Fusarium graminearum virus China 9 (FgV-ch9), which causes hypovirulence in *Fusarium graminearum*, is utilized as a tool for determining reporter gene expression (Domènech-Eres et al., 2024). The discovery of novel mycoviruses offers profound insights into viral ecology and evolution (Xie and Jiang, 2024).

In this study, we identified 10 mycoviruses from fungi associated with mango leaf spots using NGS and RNA-ligase-mediated rapid amplification of cDNA ends (RLM-RACE). Our findings include six novel viruses, three previously unreported viruses in their new hosts, and a virus strain. These findings enhance our understanding of the diversity and taxonomy of mycoviruses in mango-pathogenic fungi.

## 2 Materials and methods

### 2.1 Isolates and growth conditions

Fungal isolates were obtained from mango leaves that exhibited undefined lesion symptoms in Zhanjiang, Guangdong, China. Infected leaf segments (1 cm in diameter) were disinfected with 70% ethanol for 1 min, rinsed twice with sterile distilled water, and dried using a sterile filter paper. Five segments were placed onto potato dextrose agar (PDA; consisting of 200 g/L potato, 15 g/L agar, and 20 g/L dextrose) and incubated in the dark at 28°C for 2 days. Emerging hyphae were transferred to the fresh PDA medium. Cultures were maintained on PDA at 28°C in the dark, and fungal stocks were preserved in 25% (v/v) glycerol at −80°C.

### 2.2 dsRNA extraction, cloning, PCR amplification, and sanger sequencing

Double-stranded RNA (dsRNA) was isolated using the CF11 cellulose powder method (Morris and Dodds, 1979). Fungi were cultivated on cellophane membranes placed over PDA plates for 5 days. The hyphal mycelia (0.5 g) were harvested and used for dsRNA extraction. To remove DNA and single-stranded RNA (ssRNA), the dsRNA preparations were treated with DNase I and S1 nuclease (Takara Bio, Dalian, China) according to the manufacturer’s instructions. The purified dsRNA was separated via 1.2% agarose gel electrophoresis and visualized after staining with ethidium bromide. Target dsRNA bands were excised and purified using a FastPure Gel DNA Extraction Mini Kit (Vazyme Biotech, Nanjing, China). Cloning was performed using rPCR (Froussard, 1993).

For internal transcribed spacer (ITS) amplification, genomic DNA from dsRNA extractions was used as the template with ITS1/ITS4 primers (Glass and Donaldson, 1995). PCR conditions included initial denaturation at 95°C for 5 min, 33 cycles of denaturation at 94°C for 30 s, annealing at 58°C for 30 s, and extension at 72°C for 60 s, followed by a final extension at 72°C for 5 min (This protocol was applied to the rest of the PCR performed in this study). The purified PCR amplicons were inserted into the pMD18-T vector and then transformed into Top10 *Escherichia coli* competent cells. Three positive clones were selected from the transformed competent cells for Sanger sequencing (Tsingke Biotech, Guangzhou, China).

### 2.3 Total RNA extraction, Illumina sequencing, and analysis

The total RNA was extracted following the protocol of the NI-Sclerotinia sclerotiorum RNA reagent (NEWBIO INDUSTRY, Wuhan, China). RNA samples (~2 ng each) were pooled to a final concentration of 12 ng and sent to Suzhou Genewiz Corporation for Illumina sequencing. The raw sequence data are available in the NCBI Sequence Read Archive (SRA) under BioProject PRJNA1185921.

Raw reads were trimmed to remove adapter sequences (AAGTCGGAGGCCAAGCG-GTCTTAGGAAGACAA and AAGTCGGATCGTAGCCATGTCGTTCTGTGAGCCAAGGAGTTG) and low-quality bases using Trimmomatic v0.39 (Bolger et al., 2014). *De novo* assembly was performed using Trinity v2.8.5 (Haas et al., 2013). Assembled contigs were de-duplicated and annotated using Diamond v0.9.30.131 (Buchfink et al., 2015) against the NCBI non-redundant protein database. Virus-associated contigs were identified via BLASTn against the NCBI core nucleotide database to exclude fungal sequences.

### 2.4 Confirmation of putative mycoviruses and termini cloning

Individual total RNA of each strain was used as a template to synthesize first-strand cDNA using reverse transcriptase M-MLV (Takara, China) and random primers. Putative viral contigs were validated using RT-PCR with primer pairs listed in Supplementary Table S2.

RLM-RACE was performed to determine terminal sequences (Liu and Gorovsky, 1993). For this purpose, 100 ng of dsRNA or 10 μg of the total RNA was ligated with a PC3T7loop primer using an RNA ligase, followed by cDNA synthesis. PCR amplification was carried out with the primer PC2 and virus-specific primers (Supplementary Table S3). RACE products were cloned into pMD18-T vectors for Sanger sequencing. Nucleotide sequences were confirmed by sequencing three independent clones, and full-length virus genomes were assembled using the Sequence Assembly tool in DNAman software v7.0.2.176.

### 2.5 RNA alignment and structure prediction

Terminal identical sequences were aligned using a Multiple Alignment tool with fast alignment method of DNAMAN (version 7). Predicted panhandle structures at the termini of *NoDV1*-RNA1, 2, 3 segments, and *PmBV1* were generated using Mfold RNA structure software with default parameters (Zuker, 2003).

### 2.6 Phylogenetic analysis

Open reading frames (ORFs) were identified using the NCBI ORF Finder tool. Conserved domains were annotated using the NCBI conserved domain database^3^. Multiple sequence alignments were performed in CLUSTX with default settings and visualized with GeneDoc.

Phylogenetic trees based on RdRp sequences were constructed as described previously (Chen et al., 2024). Alignments were conducted using Mafft (Katoh and Standley, 2013), trimmed with TrimAL (Capella-Gutierrez et al., 2009), and maximum likelihood trees were built in IQ-TREE (Nguyen et al., 2015) with 1,000 bootstrap replicates. The best-fit protein substitution models were determined automatically by IQ-TREE. MEGA 7 was used for visualizing and editing phylogenetic trees.

## 3 Results

### 3.1 Identification of 10 mycoviruses in 6 fungal strains

**Figure 1:**
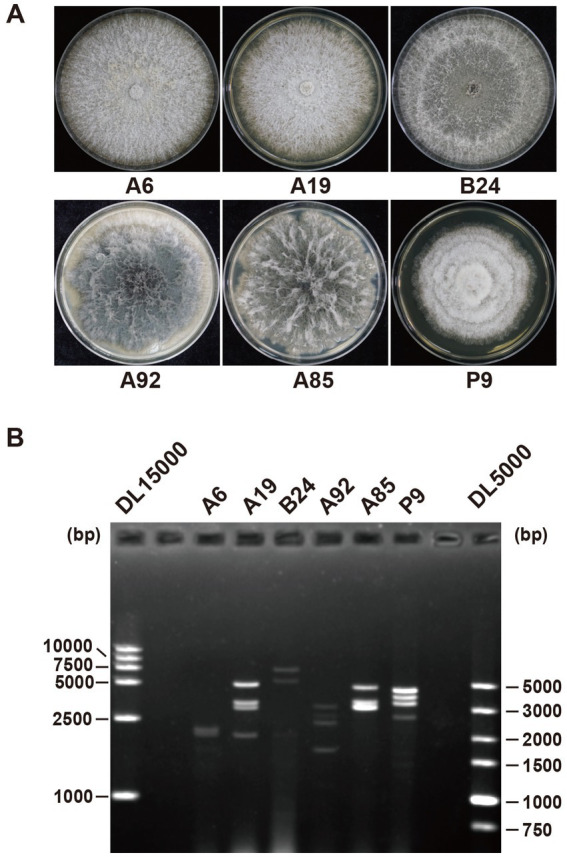
Colonies and dsRNA profiles of six fungal strains. **(A)** Colony morphology of the *Phomopsis* sp. strain A6, *Phomopsis phaseoli* strain A19, *Nigrospora oryzae* strain B24, *Botryosphaeria ramosa* strain A92, *Neofusicoccum parvum* strain A85, and *Pestalotiopsis mangiferae* strain P9. **(B)** dsRNA profiles of strains A6, A19, B24, A92, A85, and P9. The first and last lanes show markers DL15000 and DL5000, respectively (Takara, China).

Fungal strains were isolated from mango leaf spot lesions collected in the summer of 2022. Among the 300 strains tested, 6 were tested positive for dsRNA bands, which varied in both number and electrophoretic mobility (Figure 1). These strains were identified by sequencing polymerase chain reaction (PCR)-amplified internal transcribed spacer (ITS) regions and included *Phomopsis* sp. strain A6, *Phomopsis phaseoli* strain A19, *N. oryzae* strain B24, *Botryosphaeria ramosa* strain A92, *N. parvum* strain A85, and *P. mangiferae* strain P9 (Supplementary Table S1).

**Figure 2:**
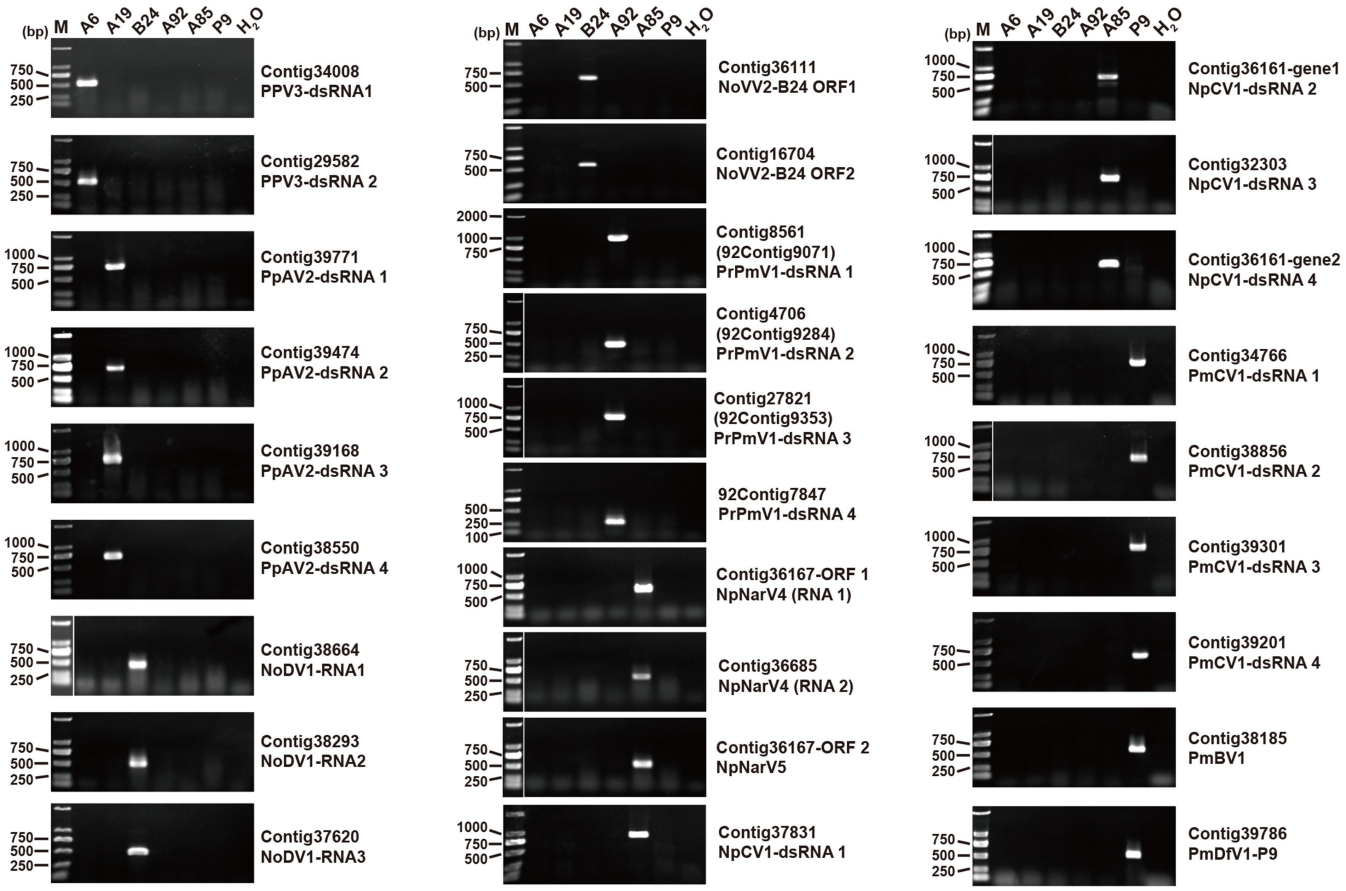
RT-PCR validation of viral contigs in six fungal strains. Primer sequences and predicted PCR product sizes are detailed in Supplementary Table S2. The electrophoresis map shows corresponding contigs and viral names on the right hand side. Lane M contains the DNA marker DL2000 (Takara, China).

The total RNA from the six strains was extracted and pooled to construct a next-generation sequencing (NGS) library. The library produced 12 GB of data, yielding 40,870,609 raw reads (SRA accession number: SRR31527143). After trimming, decontamination, and assembly, 57,272 unique contigs were identified, of which 51 were related to viral proteins based on the BLASTx analysis. Among these, 24 contigs were linked to fungal genes via BLASTn, leaving 27 contigs identified as viral segments (Table 1). The majority of the contigs contained full-length or nearly full-length coding DNA sequences (CDSs), which were confirmed by reverse transcription (RT)-PCR (Figure 2).

**Table 1:** Viral sequences in this study.

Name of viruses	Virus abbrev.	GenBank accession number	Viral full length (bp)^1^	Best match	Aa identity (%)	Family/class	Genome type
Neofusicoccum parvum narnavirus 4 (RNA1)	NpNarV4-RNA1	PQ653950	2,511	RNA-dependent RNA polymerase, Zhangzhou Narna tick virus 3 (UYL95381.1)	72	*Narnaviridae*	+ssRNA
Neofusicoccum parvum narnavirus 4 (RNA2)	NpNarV4-RNA2	PQ653952	2,271	RdRp, Downy mildew lesion associated splipalmivirus 3 (WNA22209.1)	67	*Narnaviridae*	+ssRNA
Neofusicoccum parvum Narnavirus 4 (RNA3)	NpNarV4-RNA3	PQ653951	883	Hypothetical protein, Downy mildew lesion associated splipalmivirus 4 (WNA22213.1)	69	*Narnaviridae*	+ssRNA
Pestalotiopsis mangiferae deltaflexivirus 1-P9	PmDfV1-P9	PQ653961	7,719	Neopestalotiopsis nebuloides deltaflexivirus 1 (XBR32758.1)	98	*Deltaflexiviridae*	+ssRNA
Phomopsis phaseoli alternavirus 1 (dsRNA1)	PpAV1	PQ653938	3681	RNA-dependent RNA polymerase, Diaporthe alternavirus 1 (BDQ13829.1)	97	*Alternaviridae*	dsRNA
Phomopsis phaseoli alternavirus 1 (dsRNA2)	PpAV1	PQ653939	2,678	Hypothetical protein, Diaporthe alternavirus 1 (BDQ13830.1)	98	*Alternaviridae*	dsRNA
Phomopsis phaseoli alternavirus 1 (dsRN3)	PpAV1	PQ653940	2,479	Coat protein, Diaporthe alternavirus 1 (BDQ13831.1)	96	*Alternaviridae*	dsRNA
Phomopsis phaseoli alternavirus 1 (dsRNA4)	PpAV1	PQ653941	1,699	Hypothetical protein, Diaporthe alternavirus 1 (BDQ13832.1)	81	*Alternaviridae*	dsRNA
Pestalotiopsis mangiferae chrysovirus 1 (dsRNA1)	PmCV1	PQ653957	3,480	RNA-dependent RNA polymerase, Alphachrysovirus cerasi (CAH03664.1)	59	*Chrysoviridae*	dsRNA
Pestalotiopsis mangiferae chrysovirus 1 (dsRNA2)	PmCV1	PQ653958	3,060	Putative coat protein, Alphachrysovirus cerasi (YP_001531162.1)	42	*Chrysoviridae*	dsRNA
Pestalotiopsis mangiferae chrysovirus 1 (dsRNA3)	PmCV1	PQ653959	2,789	Putative protease, Alphachrysovirus cerasi (YP_001531161.1)	48	*Chrysoviridae*	dsRNA
Pestalotiopsis mangiferae chrysovirus 1 (dsRNA4)	PmCV1	PQ653960	2,265	Hypothetical protein, Alphachrysovirus cerasi (CAH03667.1)	30	*Chrysoviridae*	dsRNA
Neofusicoccum parvum chrysovirus 2 (dsRNA1)	NpCV2	PQ653953	3,643	RNA-dependent RNA polymerase, Betachrysovirus botryosphaeriae (AJD14830.1)	97	*Chrysoviridae*	dsRNA
Neofusicoccum parvum chrysovirus 2 (dsRNA2)	NpCV2	PQ653954	2,719	Coat protein, Botryosphaeria dothidea chrysovirus 1-like (UVZ34690.1)	97	*Chrysoviridae*	dsRNA
Neofusicoccum parvum chrysovirus 2 (dsRNA3)	NpCV2	PQ653955	2,590	Hypothetical protein QK517_s3gp1, Botryosphaeria dothidea chrysovirus 1 (YP_010839425.1)	94	*Chrysoviridae*	dsRNA
Neofusicoccum parvum chrysovirus 2 (dsRNA4)	NpCV2	PQ653956	2,589	Hypothetical protein, Botryosphaeria dothidea chrysovirus 1 (YP_009353028.1)	94	*Chrysoviridae*	dsRNA
Phomopsis partitivirus 3 (dsRNA1)	PPV3	PQ653936	1807	RNA-dependent RNA polymerase, Sinodiscula camellicola partitivirus 1 (XFU40758.1)	68	*Partitiviridae*	dsRNA
Phomopsis partitivirus 3 (dsRNA2)	PPV3	PQ653937	1739	Capsid protein, Sinodiscula camellicola partitivirus 1 (XFU40757.1)	60	*Partitiviridae*	dsRNA
Botryosphaeria ramosa polymycovirus 1 (dsRNA 1)	BrPmV1	PQ653946	2,580	RNA-dependent RNA polymerase, Sclerotinia sclerotiorum tetramycovirus-1 (AWY10945.1)	47	*Polymycoviridae*	dsRNA
Botryosphaeria ramosa polymycovirus 1 (dsRNA 2)	BrPmV1	PQ653947	2,263	Hypothetical protein, Sclerotinia sclerotiorum tetramycovirus-1 (AWY10946.1)	33	*Polymycoviridae*	dsRNA
Botryosphaeria ramosa polymycovirus 1 (dsRNA3)	BrPmV1	PQ653948	2056	Methyltransferase, Sclerotinia sclerotiorum tetramycovirus-1 (AWY10947.1)	37	*Polymycoviridae*	dsRNA
Botryosphaeria ramosa polymycovirus 1 (dsRNA4)	BrPmV1	PQ653949	1,429	No match		*Polymycoviridae*	dsRNA
Nigrospora oryzae victorivirus 2-B24 (ORF1)	NoVV2-B24	PQ653942	5,161	RNA-dependent RNA polymerase, Nigrospora sphaerica victorivirus 1 (BCY26964.1)	84	*Pseudototiviridae*	dsRNA
Nigrospora oryzae victorivirus 2-B24 (ORF2)	NoVV2-B24	PQ653942	5,161	Coat protein, Nigrospora oryzae victorivirus 2 (AZP53926.1)	83	*Pseudototiviridae*	dsRNA
Pestalotiopsis mangiferae bunyavirus 1	PmBV1	PQ653962	7,190	RNA-dependent RNA polymerase, Rhizoctonia solani negative-stranded virus 4 (ALD89133.1)	46	*Bunyaviricetes*	-ssRNA
Nigrospora oryzae discovirus 1 (RNA1)	NoDV1	PQ653943	6,604	Large protein, Guyuan tick virus 1	66	*Discoviridae*	-ssRNA
Nigrospora oryzae discovirus 1 (RNA2)	NoDV1	PQ653944	1930	Non-structural protein, Leptosphaeria biglobosa negative ssRNA virus 3 (UNI72637.1)	31	*Discoviridae*	-ssRNA
Nigrospora oryzae discovirus 1 (RNA3)	NoDV1	PQ653945	1,245	Nucleocapsid protein, Penicillium roseopurpureum negative ssRNA virus 1 (YP_010840310.1)	51	Discoviridae	-ssRNA

1 Virus sequence length without the termini polynucleotide, such as poly(A) or poly(U).

The contigs detected in the *B. ramosa* strain A92, contig8561, contig4706, and contig27821, were significantly shorter than their closest viral CDS matches. To address this, a secondary NGS library was constructed using 12 ng of the total RNA from the strain A92, yielding 17 GB of data and 57,392,550 raw reads (SRA accession number: SRR31527142). The analysis revealed that the contigs 92contig9071, 92contig9284, and 92contig9395 corresponded to contig8561, contig4706, and contig27821 and included full-length CDSs. In addition, four dsRNA segments from the strain A92 were sequenced using a random RT-PCR (rPCR). Three segments corresponded to the contigs 92contig9071, 92contig9284, and 92contig9395, while the smallest segment (92contig7847) had no homology to the known genes. The terminal sequences of all identified viruses were validated using RLM-RACE (Supplementary Figures S1, S2).

### 3.2 *Botryosphaeria ramosa polymycovirus* 1

**Figure 3:**
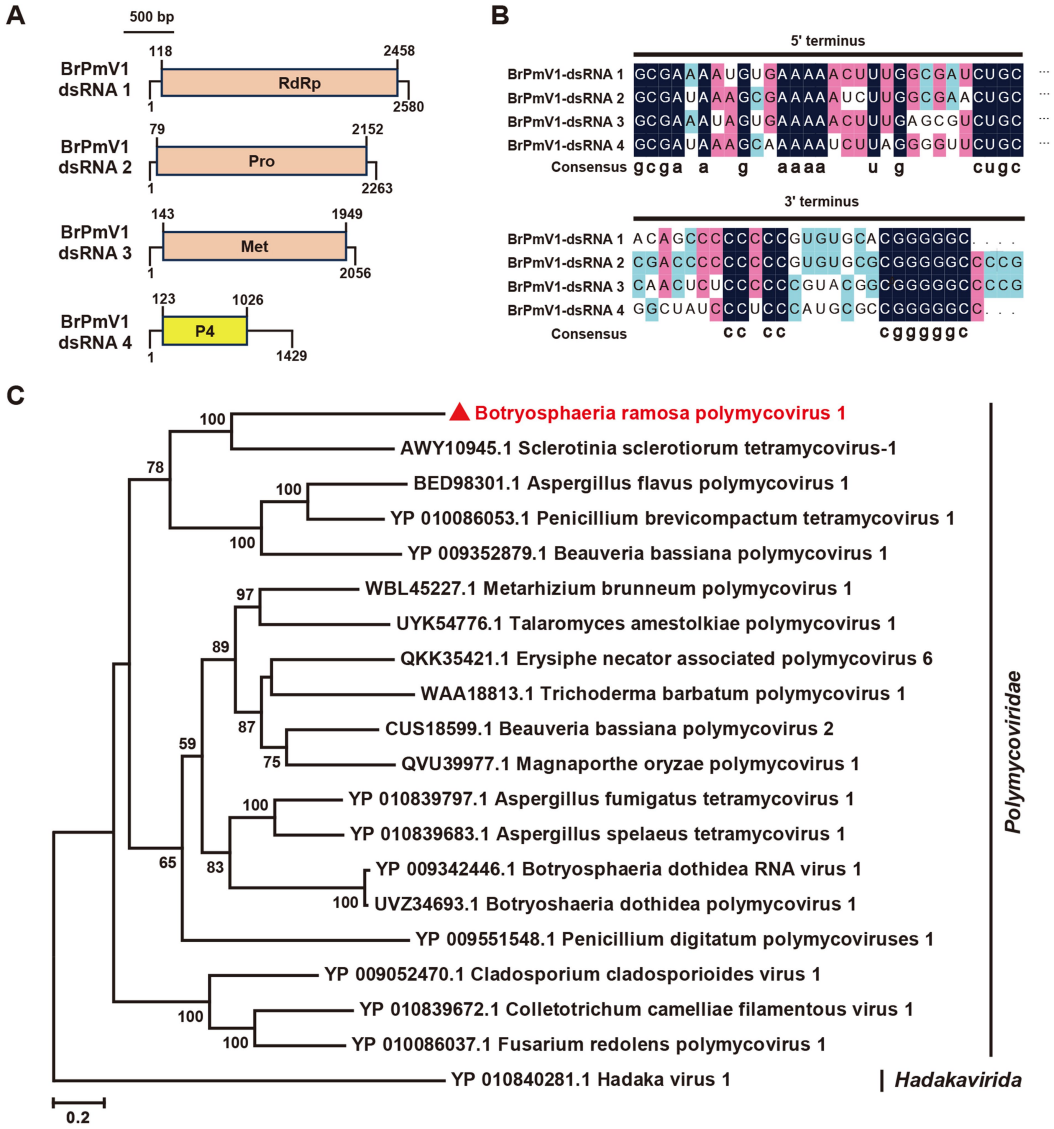
Genome organization and phylogenetic analysis of Botryosphaeria ramose polymycovirus 1 (BrPV1). **(A)** Genome schematic of BrPV1. Black lines indicate dsRNA, with colored boxes denoting ORFs. Labels include Pro (protease) and Met (methyltransferase). **(B)** Sequence similarity of the 5’ and 3’ terminal regions of BrPV1 RNA1–4. **(C)** Phylogenetic tree of *Polymycoviridae* viruses, based on RdRp sequences, constructed using the maximum likelihood method.

The Polymycoviridae family comprises multipartite dsRNA viruses with genome sizes ranging from 7.5 to 12.5 kb, typically organized in four to eight segments (Kotta-Loizou et al., 2022). Recent findings suggest that some members form filamentous virions, redefining their morphology (Jia et al., 2017; Kotta-Loizou et al., 2022). In this study, we identified the contigs 92contig9071, 92contig9284, and 92contig9395 in the *B. ramosa* strain A92, which shared 47, 33, and 37% identity, respectively, with proteins from Sclerotinia sclerotiorum tetramycovirus-1 (SsTmV1; Table 1). These segments, collectively named *Botryosphaeria ramosa polymycovirus* 1 (BrPmV1), formed a genome totaling 8,328 bp. The first segment (2,580 bp) encoded the RNA-dependent RNA polymerase (RdRp); the second (2,263 bp) encoded a serine protease-like protein; the third (2,056 bp) encoded methyltransferase; and the fourth (1,429 bp) encoded an uncharacterized protein (Figure 3A). All four segments shared identical GC-rich terminal sequences (Figure 3B). The phylogenetic analysis categorized BrPmV1 within the Polymycoviridae family (Figure 3C), highlighting its contribution to the diversity of this viral group.

### 3.3 A tri-segmented narnavirus in *Neofusicoccum parvum*

**Figure 4:**
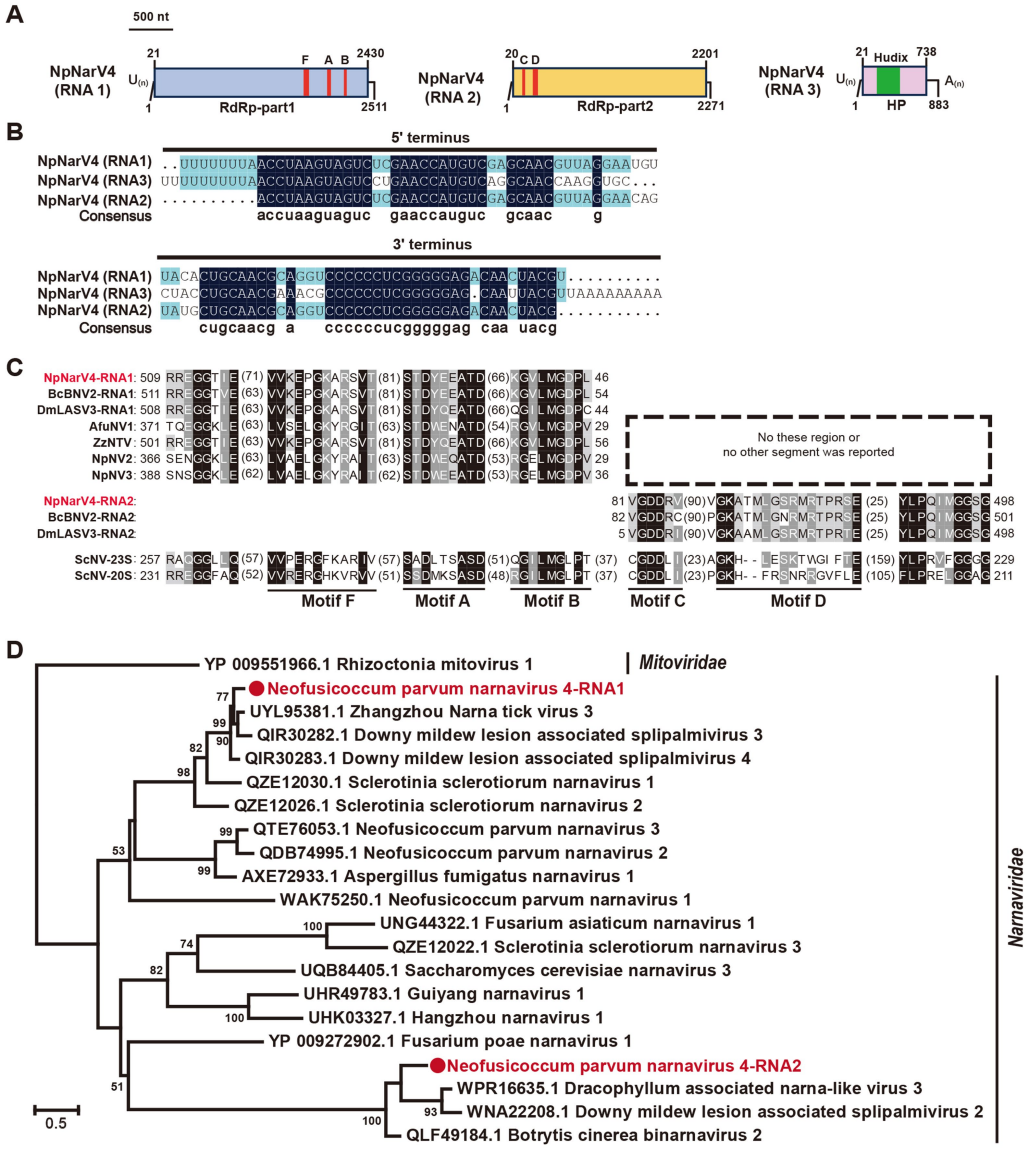
Genome organization and phylogenetic analysis of Neofusicoccum parvum narnavirus 4 (NpNarV4). **(A)** Genome schematics for NpNarV4 RNA1-3. Black lines indicate dsRNA, with motifs (F, A, B, C, D) and ORFs outlined in colored boxes. **(B)** Sequence similarity of 5’ and 3’ terminal regions of NpNarV4 RNA1-3. **(C)** Alignment of RdRp motifs from *Narnavirus* genus viruses. Accession numbers and virus names are listed in Table S5. **(D)** Phylogenetic tree of *Narnaviridae* viruses, based on RdRp sequences, using the maximum likelihood method.

Narnaviridae is a family of simple, un-encapsidated +ssRNA viruses with genomes ranging from 2.3 to 2.9 kb, typically encoding a single RdRp (Hillman and Cai, 2013). Recent studies have described multi-segmented narnaviruses (Daghino et al., 2024; Muñoz-Suárez et al., 2024). In this study, we identified a unique tri-segmented narnavirus in the *N. parvum* strain A85, designated Neofusicoccum parvum narnavirus 4 (NpNarV4). Contig36167, containing two open reading frames (ORFs), was detected as two distinct segments (contig36167orf1 and contig36167orf2; Figure 2). No amplification was achieved using primers spanning the two ORFs. An additional contig, contig36685, encoding a hypothetical protein was detected in this strain. The full-length sequences of the three segments, designated NpNarV4-RNA1, RNA2, and RNA3, were 2,511 nt, 2,271 nt, and 883 nt, respectively, excluding poly(A) or poly(U) tails (Figure 4A). All segments shared identical terminal sequences, with RNA1 containing a poly(U) at the 5′ termini and RNA3 also containing a poly(U) at the 5′ terminus and a poly(A) at the 3′ termini (Figure 4B).

Proteins encoded by NpNarV4-RNA1, RNA2, and RNA3 shared 72, 67, and 69% identities, respectively, with Zhangzhou narna tick virus 3, Downy mildew lesion-associated splipalmivirus 3, and Downy mildew lesion-associated splipalmivirus 4. Five conserved RdRp motifs were identified across RNA1 and RNA2 (Figure 4C). Interestingly, RNA3 encoded a hypothetical protein related to NUDIX hydrolase of *Streptomyces* sp. (22% identity; DELTA-BLAST).

Given its novel host and unique genetic features, NpNarV4 represents a new member of the Narnaviridae family. The phylogenetic analysis of RdRp motifs revealed distinct clustering of RdRp-part1 and RdRp-part2, differing from Neofusicoccum parvum narnavirus 1, 2, and 3 (Figure 4D), underscoring the genetic and evolutionary complexity of the family.

### 3.4 Two Bunya-like mycoviruses

**Figure 5:**
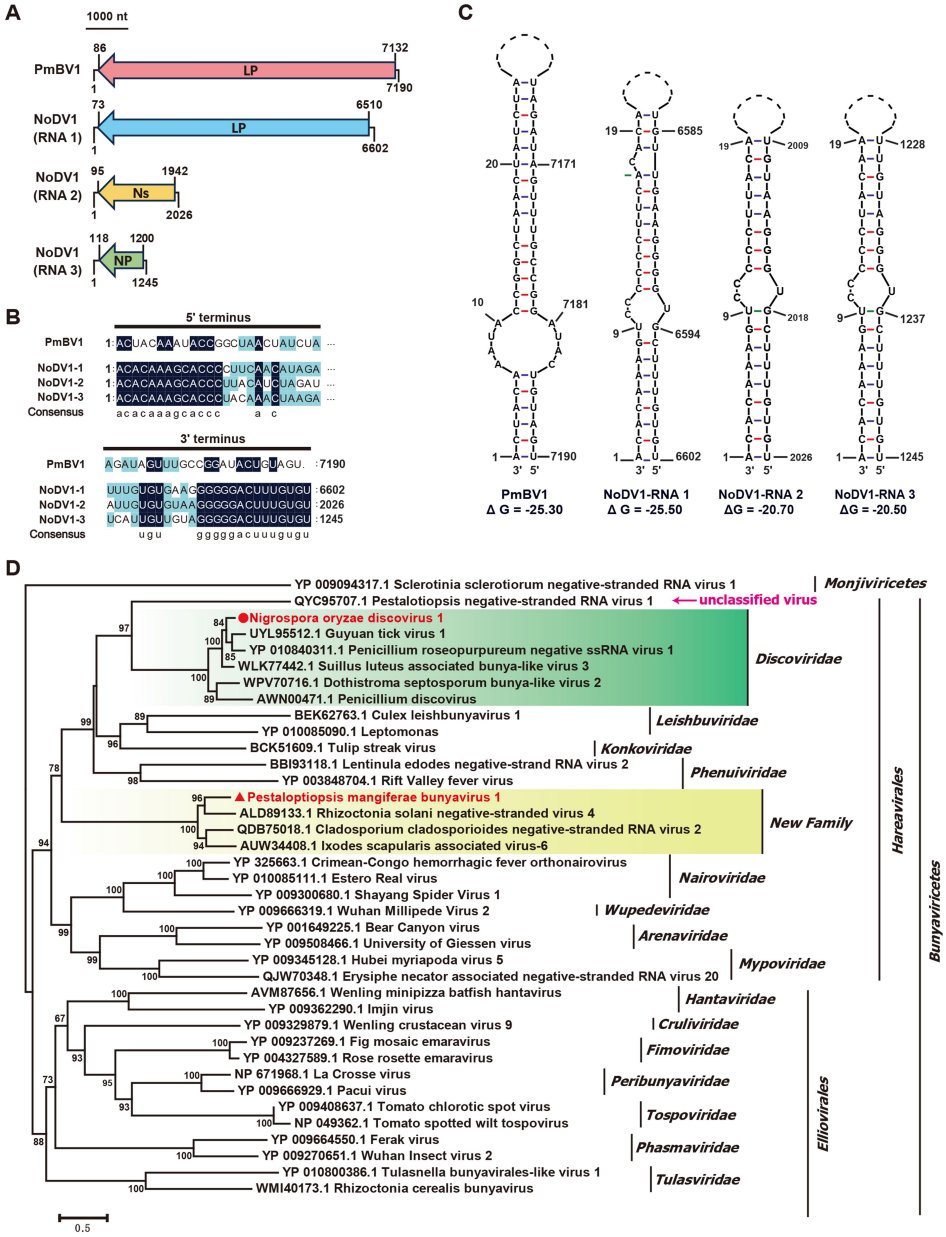
Genome organization and phylogenetic analysis of Nigrospora oryzae discovirus 1 (NoDV1) and Pestalotiopsis mangiferae bunyavirus 1 (PmBV1). Black lines represent genomic dsRNA. Colored boxes represent the ORFs encoded by positive-strand viral RNAs. LP, large protein; Ns, nonstructure protein; NP, nucleocapsid protein. **(A)** Genome schematics for NoDV1 RNA1 -3 and PmBV1. Black lines indicate dsRNA, with ORFs shown in colored boxes. **(B)** Sequence similarity of 5’ and 3’ terminal regions of NoDV1 RNA1 -3 and PmBV1 segments. **(C)** Complementarity analysis of the 5’ and 3’ terminal regions of NoDV1 RNA1 -3 and PmBV1, showing potential panhandle structures formed by inverted complementarity, as predicted using Mfold RNA structure software. **(D)** Phylogenetic tree of *Bunyaviricetes viruses*, based on RdRp sequences, using the maximum likelihood method.

The Class *Bunyaviricetes* includes two major orders, *Elliovirales* (e.g., *Cruliviridae, Fimoviridae, Hantaviridae*) and *Hareavirales* (e.g., *Arenaviridae, Discoviridae, Leishbuviridae*), comprising hundreds of viruses that infect humans, animals, plants, and fungi (Kuhn et al., 2024). In our study, the *N. oryzae* strain B24 yielded three contigs, contig38664, contig38293, and contig37620, that encoded bunya-like proteins related to a large protein (LP), non-structural protein (NS), and nucleocapsid protein (NP) (Figure 2, Table 1). These segments, designated *Nigrospora oryzae discovirus 1* (NoDV1), were 6,602 nt, 2,026 nt, and 1,245 nt in length, respectively (Figure 5A). NoDV1-RNA1 encoded a large protein sharing 66% identity with Guyuan tick virus 1 (GtV1, an insect virus). NoDV1-RNA2 encoded a non-structural protein sharing 31% identity with Leptosphaeria biglobosa negative ssRNA virus 3 (LbNSRV3). NoDV1-RNA3 encoded nucleocapsid protein sharing 51% identity with Penicillium roseopurpureum negative ssRNA virus 1 (PrNSRV1). Notably, the 5′ and 3′ terminal sequences of each NoDV1 segment were identical (Figure 5B).

In the *P. mangiferae* strain P9, contig38185 encoded an RdRp related to *Rhizoctonia solani negative-stranded virus 4* (RsNSV4) with 46% identity, which we named *Pestalotiopsis mangiferae bunyavirus 1* (PmBV1) (Figure 2, Table 1). The full length of PmBV1 was 7,190 nt, with complementary 3′ and 5′ terminal structures similar to those observed in NoDV1 (Figure 5C). The phylogenetic analysis categorized NoDV1 within the family *Discoviridae* and PmBV1 among unclassified viruses in the order *Hareavirales* (Figure 5D). These findings revealed NoDV1 and PmBV1 as novel bunya-like mycoviruses in fungi.

### 3.5 Two viruses related to *Chrysovirus*

**Figure 6:**
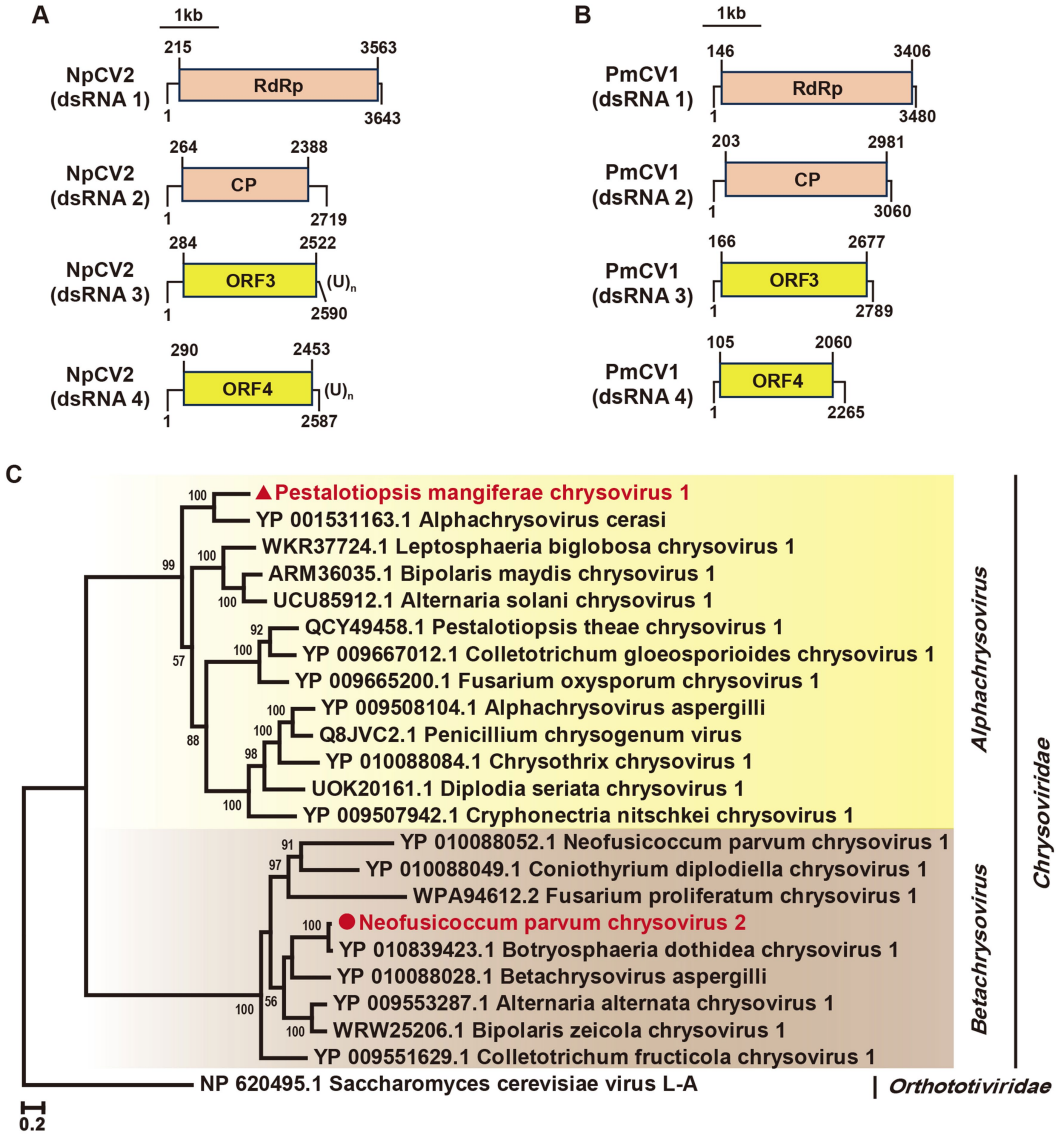
Genome organization and phylogenetic analysis of Neofusicoccum parvum chrysovirus 2 (NpCV2) and *Pestalotiopsis mangiferae chrysovirus* 1 (PmCV1). **(A)** Schematic representation of NpCV2. **(B)** Schematic representation of PmCV1 genomes. Black lines represent dsRNA, with ORFs shown in colored boxes. **(C)** Phylogenetic tree of *Chrysoviridae* viruses, based on RdRp sequences, constructed using the maximum likelihood method.

The *Chrysoviridae* family consists of dsRNA viruses typically encapsidated into four segments ranging from 2.5 to 3.6 kb (Kotta-Loizou et al., 2020). We identified two novel chrysoviruses in the *N. parvum* strain A85 and the *P. mangiferae* strain P9, respectively. In strain A85, contigs 37,831, 36,161-gene1, 36,161-gene2, and 32,303 corresponded to *Botryosphaeria dothidea chrysovirus 1* (BdCV1) (Table 1) and have been designated *Neofusicoccum parvum chrysovirus 2* (NpCV2). These segments are 3,643 bp, 2,719 bp, 2,590 bp, and 2,589 bp in length, respectively (Figure 6A). Notably, dsRNA3 and dsRNA4 contained poly(U) sequences at their 3′ termini. Predicted ORFs exhibited high identities (97, 97, 94, and 94%) with BdCV1 proteins, identifying NpCV2 as a novel host-associated chrysovirus.

In strain P9, contigs 34,766, 38,856, 39,201, and 39,301 corresponded to *Alphachrysovirus cerasi* and have been named *Pestalotiopsis mangiferae chrysovirus 1* (PmCV1). These four segments measured 3,480 bp, 3,060 bp, 2,789 bp, and 2,265 bp in length, each encoding a single predicted ORF (Figure 6B). The proteins of PmCV1 exhibited 59, 42, 48, and 30% identities with those of *Alphachrysovirus cerasi, respectively* (Table 1).

The phylogenetic analysis revealed that NpCV2 clustered with the genus *Betachrysovirus*, while PmCV1 aligned with the genus *Alphachrysovirus* (Figure 6C). Collectively, our findings proposed PmCV1 as a novel member of the *Chrysoviridae family*.

### 3.6 *Phomopsis partitivirus* 3

The *Partitiviridae family*, widespread among plants, fungi, and protozoa, comprises five recognized genera: *Alphapartitivirus, Betapartitivirus, Deltapartitivirus, Gammapartitivirus,* and *Cryspovirus* (Vainio et al., 2018). Some viruses, however, remain unclassified within these genera, leading to proposals for two additional genera: *Epsilonpartitivirus* and *Zetapartitivirus* (Zhu et al., 2024). Previously, *Phomopsis asparagi partitivirus 1* (PaPV1) and *Phomopsis vexans partitivirus 1* (PvPV1) were classified under *Gammapartitivirus* and *Deltapartitivirus*, respectively^2^.

**Figure 7:**
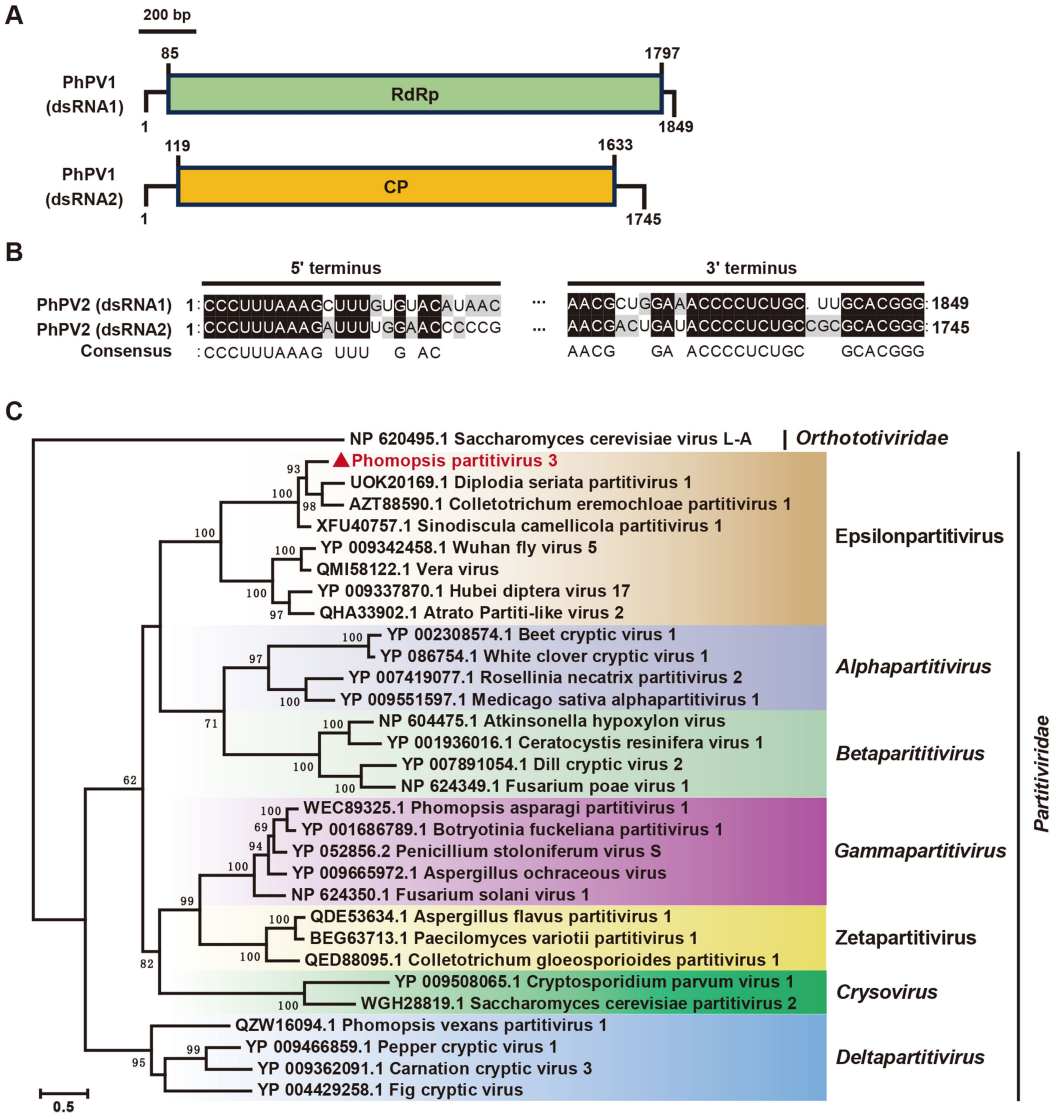
Genome organization and phylogenetic analysis of Phomopsis partitivirus 3 (PhPV3). **(A)** Schematic diagram of the PhPV3 genome. Black lines indicate dsRNA, while colored boxes represent ORFs encoded by positive-strand viral RNAs. **(B)** Sequence similarity analysis of the 5’ and 3’ terminal regions of PhPV3 dsRNA1 and dsRNA2. **(C)** Phylogenetic tree of viruses in the *Partitiviridae* family, based on RdRp sequences, constructed using the maximum likelihood method.

In our study, contig 34,008 and contig 29,582 were detected in the *Phomopsis* sp. strain A6, exhibiting 68 and 60% identities, respectively, with the RdRp and coat protein (CP) of *Sinodiscula camellicola partitivirus 1* (ScPV1) (Figure 2, Table 1). These were designated as *Phomopsis partitivirus 3* (PhPV3). The two dsRNA segments of PhPV3 measured 1,849 bp and 1,745 bp in length, respectively, and shared identical terminal sequences (Figures 7A,B). The phylogenetic analysis categorized PhPV3 within the proposed genus *Epsilonpartitivirus*, distinctly separate from PaPV1 and PvPV1 (Figure 7C). PhPV3, found in a novel host, represents a new member of the proposed genus *Epsilonpartitivirus*.

### 3.7 Discovery of three additional viruses

**Figure 8:**
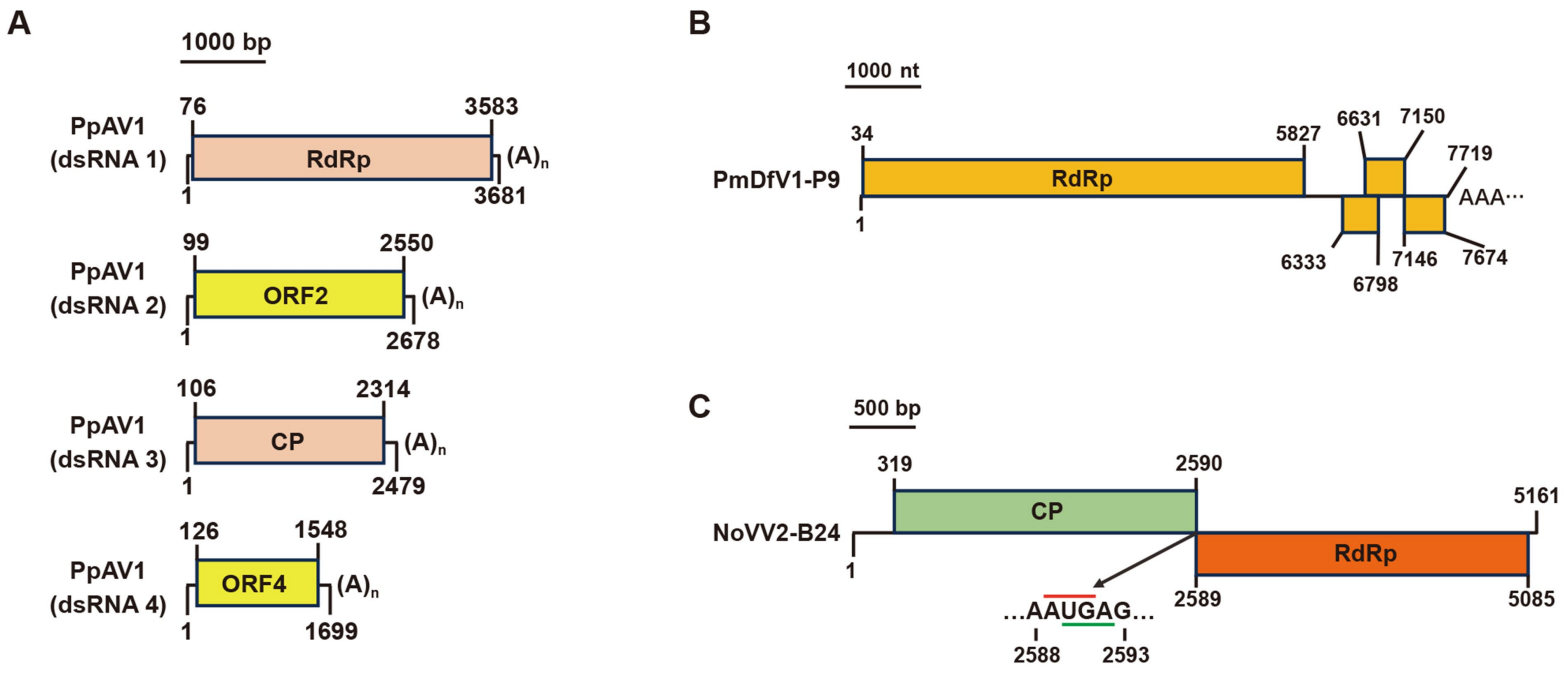
Genome organization of **(A)** Phomopsis phaseoli alternavirus 1 (PpAV1), **(B)** *Nigrospora oryzae* victorivirus 2-B24 (NoVV2-B24), and **(C)** *Pestalotiopsis mangiferae deltaflexivirus* 1-P9 (PmDfV1-P9). Black lines indicate dsRNA. Colored boxes represent ORFs, while red and green lines denote start and stop codons, respectively.

Our study identified three additional mycoviruses with significant amino acid similarity to known viruses. In the *P. phaseoli* strain A19, contig 39,771, contig 39,474, contig 39,168, and contig 38,550 encoded four predicted proteins sharing identities of 97, 98, 96, and 81%, respectively, with *Diaporthe alternavirus 1* (DaV1). Since *Phomopsis* represents the asexual state of *Diaporthe*, and no spores of strain A19 were observed on PDA plates, we designated this virus as *Phomopsis phaseoli alternavirus 1* (PpAV1). Similar to DaV1, the genome RNAs of PpAV1 contained poly(A) tails (Figure 8A).

In the *P. mangiferae* strain P9, contig 39,786 encoded an RdRp sharing 98% identity with *Neopestalotiopsis nebuloides deltaflexivirus 1*. This virus was named *Pestalotiopsis mangiferae deltaflexivirus 1-P9* (PmDfV1-P9). The PmDfV1-P9 genome encoded four predicted ORFs and featured a polyA tail at its 3′ terminus (Figure 8B).

Finally, in the *N. oryzae* strain B24, contig 16,704 and contig 36,111 encoded an RdRp and a coat protein (CP), sharing 84 and 83% identities, respectively, with *Nigrospora oryzae victorivirus 2* (NoVV2). To confirm the sequence linkage between contig16704 and contig36111, primers vicF2866 (located in Contig36111) and vicR4131 (in Contig16704) were designed, respectively (Supplementary Table S4), and amplification results confirmed the full-length sequence of *Nigrospora oryzae victorivirus 2-B24* (NoVV2-B24) (Supplementary Figure S3). The complete genome of NoVV2-B24 spans a length of 5,161 bp and features two large overlapping ORFs (Figure 8C).

## 4 Discussion

This is the first study to document the presence of 10 mycoviruses in 6 fungi that are associated with mango leaf spots in China. Using NGS and rPCR, 27 viral sequences were efficiently identified from 6 dsRNA-containing fungal strains. A total of 10 mycoviruses were classified into 8 distinct lineages: *Alternaviridae*, *Chrysoviridae*, *Partitiviridae*, *Polymycoviridae*, *Orthototiviridae*, *Deltaflexiviridae*, *Narnaviridae*, and *Bunyaviricetes*. With the exception of NoVV2-B24, which is a viral strain of NoVV2, the other nine viruses exhibited less than 60% amino acid sequence identity with their closest known references or were discovered in novel hosts. This suggests either significant divergence or association with previously unidentified viruses.

In this study, the initial NGS pool comprised only six fungal strains—a relatively limited number of mixed species. Although a majority of the viral contigs contained complete coding sequences (CDSs), a few contigs, such as contig 8,561, contig 4,706, and contig 27,821, were considerably shorter than the related genomic RNAs of polymycoviruses. To address this, a second NGS pool was prepared using the total RNA from the strain A92, which produced lengthy contigs (92contig9071, 92contig9284, and 92contig9395) with complete CDSs of BrPmV1. Additionally, rPCR revealed that the smallest dsRNA band (1.5 kb) from the strain A92 corresponded to BrPmV1 RNA4. Notably, when we used the sequence of BrPmV1 RNA4 for BLASTn against the two NGS assemblies, no matching contig was found in the mixed-fungi NGS pool. However, the second NGS assembly contained 92contig7847, which encoded the entire CDS. These findings indicate that pooling multiple fungal species in NGS experiments may compromise the integrity of specific viral sequences. NGS of individual strains and rPCR targeting specific fragments could improve the integrity and accuracy of virome investigation.

Polymycoviruses, which possess the characteristics of both dsRNA and + ssRNA viruses, as well as encapsidated and capsidless RNA viruses, are known to influence host phenotypes (Wang et al., 2023). For instance, *Metarhizium anisopliae polymycovirus 1* enhances host growth, conidiation, and UV-B sensitivity (Wang et al., 2023), while *Aspergillus fumigatus polymycovirus 1* slows host growth and increases susceptibility to stressors such as high temperature, Congo red, and hydrogen peroxide (Sass et al., 2023). In this study, dsRNA1, dsRNA2, and dsRNA3 of BrPmV1 showed low sequence identities with SsTmV1. The smallest BrPmV1 dsRNA4 appears to encode a novel protein with no homolog in the known databases. Whether BrPmV1 forms filamentous particles remains unclear. Importantly, BrPmV1 is the first polymycovirus identified in *B. ramosa* and appears to be phylogenetically distinct from other polymycoviruses infecting *Botryosphaeria* species.

The *Narnaviridae* family is characterized by simple RNA viruses that typically encoded only one RdRp protein, as seen in the *Saccharomyces 20S RNA narnavirus* (ScNV-20S) and *Saccharomyces 23S RNA narnavirus* (ScNV-23S). Recent studies have revealed narnaviruses with multiple segments, such as *splipalmiviruses*, where the RdRp palm domain is divided across separate genomic segments (Sato et al., 2022; Daghino et al., 2024). In this study, the five RdRp motifs of NpNarV4 were divided into two segments. Two parts of RdRp were also grouped into two separate phylogenetic branches, which implied their evolutionary independence; but how the two RdRp parts supply the replication of NpNarV4 remain to be explored. Some narnaviruses also possessed a third segment encoding hypothetical proteins, such as *Magnaporthe oryzae narnavirus 1* and *Oidiodendron maius splipalmivirus 1* (Chiba et al., 2021; Daghino et al., 2024). Intriguingly, the RNA3 of *Botrytis cinerea narnavirus 1* (BcNV1) encoded a hypothetical protein homologous to the envelope glycoproteins of herpesviruses and the capsid proteins of the white spot syndrome virus (Muñoz-Suárez et al., 2024). NpNarV4 RNA3 encoded a protein featuring a NUDIX hydrolase 26 domain (accession number cd04685). Similar domains have been observed in the proteins of *African swine fever virus* (ASFV), *Lentinula edodes mycovirus HKB* (LeVHKB), *Rhizoctonia fumigate virus 1* (RfV1), and *Thelephora terrestris virus 1* (TtV1) (Cartwright et al., 2002; Magae, 2012; Li et al., 2015; Petrzik et al., 2016). NUDIX hydrolases are known for their “housekeeping” functions such as removing harmful metabolites (McLennan, 2006). The g5R protein of ASFV (dsDNA virus) regulates viral morphogenesis involving diphosphoinositol polyphosphate-mediated membrane trafficking (Cartwright et al., 2002). LeVHKB, RfV1, and TtV1 are single-segment dsRNA mycoviruses encoding large hypothetical proteins (162, 198, 202 kDa) with NUDIX motifs. The discovery of NpNarV4 as the first +ssRNA virus encoding a NUDIX domain underscores its novelty and potential biological significance.

The first evidence of fungal negative-stranded RNA virus was identified in the phytopathogenic ascomycete *Erysiphe pisi* (Kondo et al., 2013), followed by *Sclerotinia sclerotiorum negative-stranded RNA virus 1* (SsNSRV1) (Liu et al., 2014). Subsequent discoveries included a bunya-like mycovirus with a single-segment genome (Donaire et al., 2016) and multipartite bunya-like mycoviruses in *Lentinula edodes*, *Valsa mali*, and *Sclerotinia sclerotiorum* (Lin et al., 2019; Huang et al., 2023; Dai et al., 2024). *Pestalotiopsis negative-stranded RNA virus 1* (PNSRV1), an unclassified bunyavirus, was identified in *Pestalotiopsis* sp. (Chen et al., 2021). Phylogenetic analyses revealed that PmBV1 and NoDV1 belong to the order *Hareavirales* within the class *Bunyaviricetes*. Independent from PNSRV1, PmBV1 formed a distinct branch with *Rhizoctonia solani negative-stranded virus 4* (RsNSV4), *Cladosporium cladosporioides negative-stranded RNA virus 2* (CcNSRV2), and *Ixodes scapularis associated virus-6* (IsAV-6), suggesting the potential existence of a new family within *Hareavirales*. RdRp, Ns, and NP of NoDV1 were most closely related to GtV1, LbNSRV3, and PrNSRV1, respectively. Notably, NoDV1 represents the first discovirus identified in *N. oryzae*. These findings demonstrate the diversity and prevalence of negative-stranded RNA viruses in the fungal kingdom.

In this study, we identified two chrysoviruses. PmCV1 is the first chrysovirus discovered in *P. mangiferae*, forming a distinct phylogenetic branch alongside *Alphachrysovirus cerasi*. It also represents the second chrysovirus found in Pestalotioid fungi. Meanwhile, NpCV2, although closely related to *BdCV1*, was identified as a new host. Notably, the P3 protein encoded by *BdCV1* functions as a silencing suppressor, slowing growth and reducing the virulence of *B. dothidea* (Li et al., 2023). Whether P3 protein of NpCV2 exhibits similar silencing suppressor activity and impacts the phenotypes of *N. parvum* remains to be explored.

The phylogenetic analysis revealed that PhPV3 clustered with members of the proposed *Epsilonpartitivirus* family, which includes both mycoviruses (e.g., *Diplodia seriata partitivirus 1*, *Colletotrichum eremochloae partitivirus 1*, and *ScPV1*) and insect viruses (e.g., *Wuhan fly virus 5*, *Vera virus*, *Hubei diptera virus 17*, and *Atrato partiti-like virus 2*). However, the mycovirus and insect virus clades within this family remained distinct, suggesting independent evolutionary trajectories across different hosts.

The genomic analysis of several positive-stranded RNA viruses revealed 3′-poly(A) and 5′-poly(U) tracts (van Ooij et al., 2006). Our study identified the presence of poly(U) or poly(A) in partial viral genome termini. Specifically, we observed 3′-poly(A) or poly(U) sequences in the genome termini of NpNarV4 and PpAV1, while the RNA3 and RNA4 segments of NpCV2 exhibited 3′-poly(U) tracts, respectively. Such sequences, previously identified in the hepatitis C virus (+ssRNA), are known to play critical roles in viral replication (You and Rice, 2008). The presence of poly(U) tracts in NpCV2 highlights the need for further investigation into their potential functions in viral replication. In addition, our study identified five multipartite viruses—PhPV3, PpAV1, BrPmV1, NpNarV4, and NoDV1—with identical terminal sequences. This finding underscores the importance of cis-acting elements in regulating viral replication processes and warrants deeper functional studies.

## 5 Conclusion

Our research significantly advances the field of mycovirology, identifying six novel mycoviruses: BrPmV1, NpNarV4, NoDV1, PmBV1, PmCV1, and PhPV3. These discoveries enrich the known diversity of mycoviruses and their potential ecological roles in fungal hosts. Additionally, we documented four other viruses—PpAV1, NpCV2, and PmDfV1-P9 in new hosts and NoVV2-B24 as a new viral strain. These findings suggest that mycoviruses not only adapt to new hosts but also evolve into novel strains, offering insights into viral evolution. Beyond their evolutionary implications, our discoveries highlight the potential biocontrol applications of mycoviruses against plant pathogenic fungi. Exploring the diversity, host adaptability, and molecular mechanisms of these mycoviruses opens promising avenues for advancing agricultural, ecological, and virological research.

## Data Availability

The datasets presented in this study can be found in online repositories. The names of the repository/repositories and accession number(s) can be found in the article/Supplementary material.

## Footnotes

^1^ https://ictv.global/vmr

^3^ http://www.ncbi.nlm.nih.gov/Structure/cdd/wrpsb.cgi

^2^ www.ncbi.nlm.nih.gov/Taxonomy/

## Supplementary material

SUPPLEMENTARY FIGURE S1 RLM-RACE amplification of viral termini (Part 1). RACE amplification products confirm viral termini of **(A)** NpNarV4, **(B)** BrPmV1, **(C)** NoDV1, and **(D)** PmBV1. Specific primers and target termini are labeled. Primer sequences are in Supplementary Table S3. Red arrows indicate target PCR products. Lane M: DNA marker DL2000 (Takara).

SUPPLEMENTARY FIGURE S2 RLM-RACE amplification of viral termini (Part 2). RACE products confirm termini of **(A)** PpAV1, **(B)** PhPV3, **(C)** PmCV1, **(D)** NoVV2-B24, **(E)** NpCV2, and **(F)** PmDfV1-P9. Specific primers and target termini are labeled. Primer sequences are in Supplementary Table S3. Red arrows indicate target PCR products. Lane M: DNA marker DL2000 (Takara).

SUPPLEMENTARY FIGURE S3 RT-PCR validation of the gap between contigs 16704 and 36111. Primer vicF2866 targets contig36111, and primer vicR4131 targets contig16704. Primer sequences are listed in Supplementary Table S4. Lane M shows DNA marker DL2000 (Takara).
