# Supplementary figures and images for: Discovery of novel mycoviruses from fungi associated with mango leaf spots
Source: Front Microbiol. 2025 Feb 26;16:1545534. doi: 10.3389/fmicb.2025.1545534 (PMC11897279; doi:10.3389/fmicb.2025.1545534)

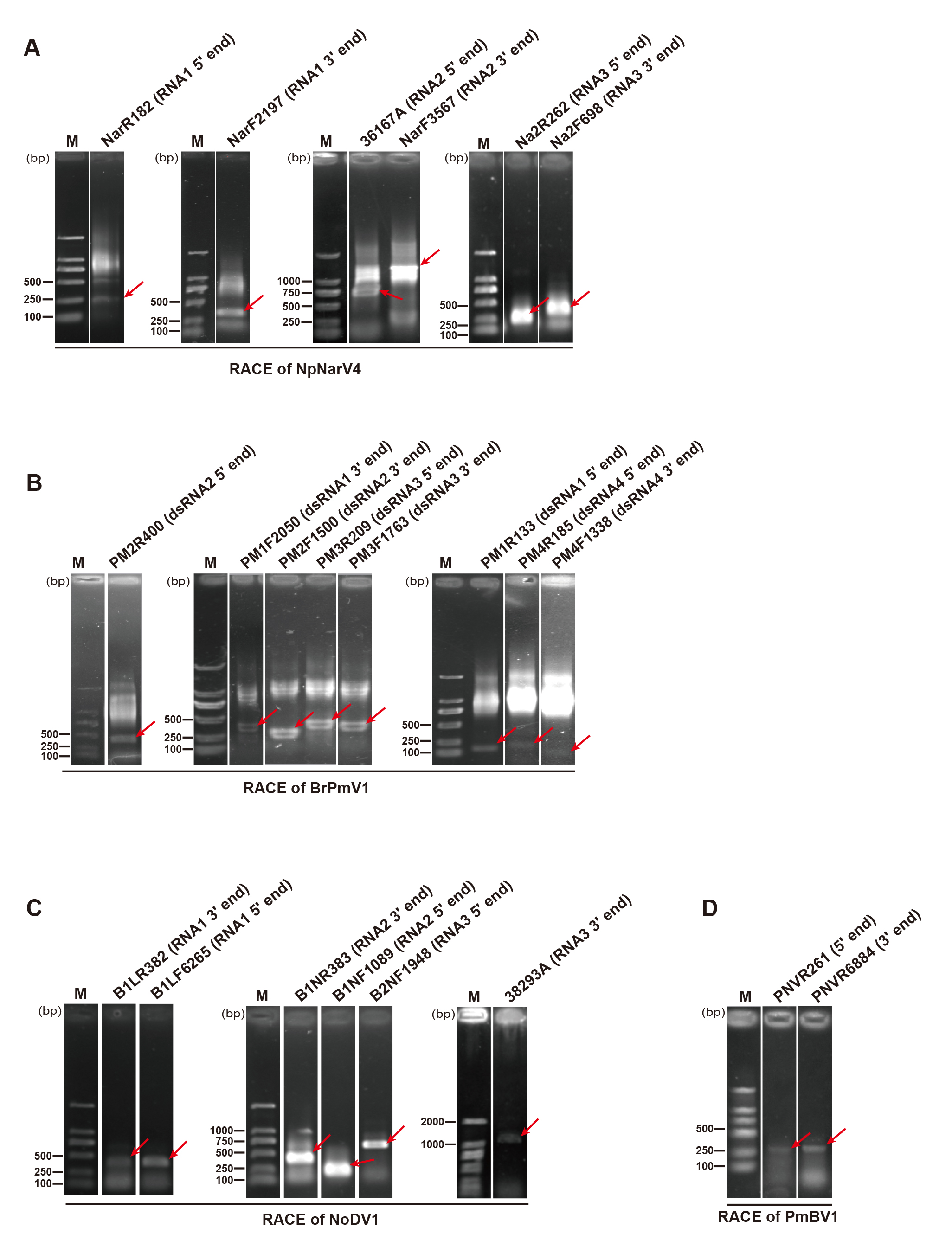

Supplement: SUPPLEMENTARY FIGURE S1 — RLM-RACE amplification of viral termini (Part 1). RACE amplification products confirm viral termini of (A) NpNarV4, (B) BrPmV1, (C) NoDV1, and (D) PmBV1. Specific primers and target termini are labeled. Primer sequences are in Supplementary Table S3. Red arrows indicate target PCR products. Lane M: DNA marker DL2000 (Takara). [file Image_1.jpeg]

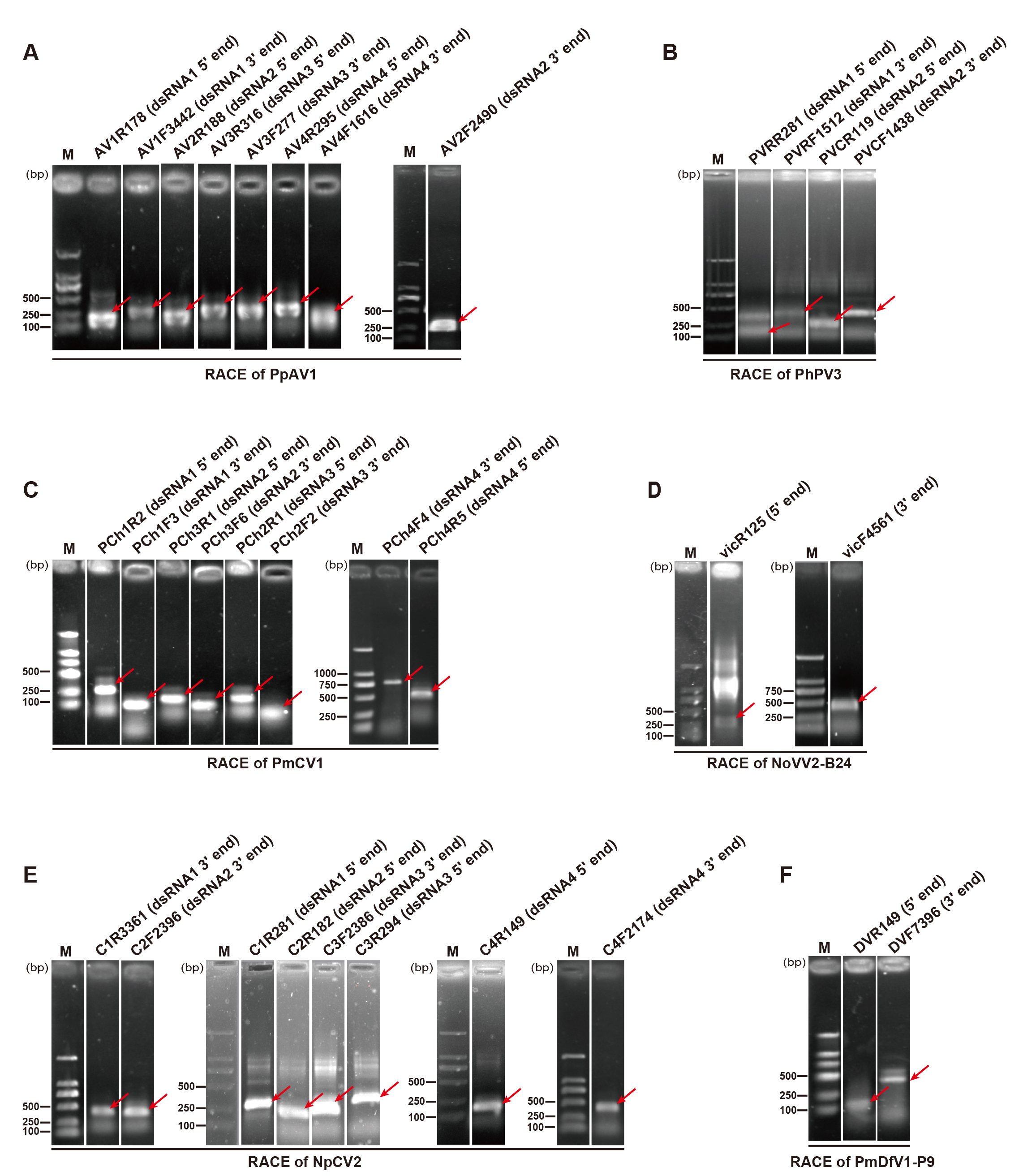

Supplement: SUPPLEMENTARY FIGURE S2 — RLM-RACE amplification of viral termini (Part 2). RACE products confirm termini of (A) PpAV1, (B) PhPV3, (C) PmCV1, (D) NoVV2-B24, (E) NpCV2, and (F) PmDfV1-P9. Specific primers and target termini are labeled. Primer sequences are in Supplementary Table S3. Red arrows indicate target PCR products. Lane M: DNA marker DL2000 (Takara). [file Image_2.jpeg]

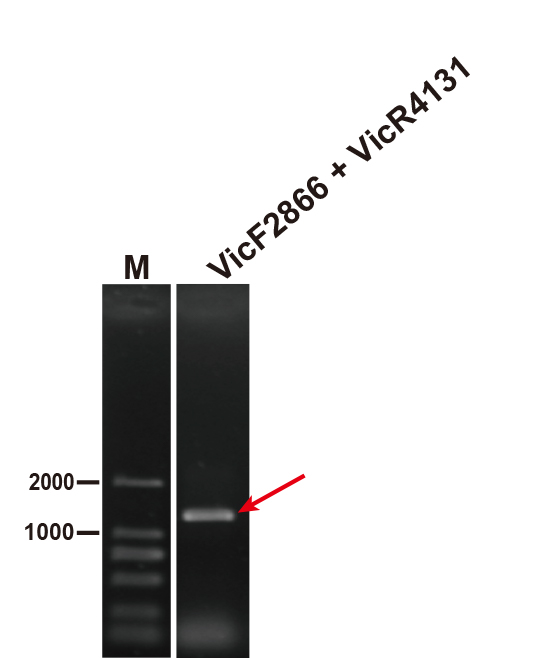

Supplement: SUPPLEMENTARY FIGURE S3 — RT-PCR validation of the gap between contigs 16704 and 36111. Primer vicF2866 targets contig36111, and primer vicR4131 targets contig16704. Primer sequences are listed in Supplementary Table S4. Lane M shows DNA marker DL2000 (Takara). [file Image_3.jpeg]
