# Supplementary material for: Discovery of novel mycoviruses from fungi associated with mango leaf spots
Source: Front Microbiol. 2025 Feb 26;16:1545534. doi: 10.3389/fmicb.2025.1545534 (PMC11897279; doi:10.3389/fmicb.2025.1545534)
Supplement: Supplementary file 4 [file Table_1.docx]

Table S1 Genbank accession number of strains’ ITS

| Strain | Genbank accession number  (ITS sequence) | Best match | Identity (%) | Family |
| --- | --- | --- | --- | --- |
| A6 | PQ614816 | *Phomopsis* sp. S12 RXT-2013 (KC193052.1) | 99.46 | *Valsaaceae* |
| A19 | PQ614817 | *Phomopsis phaseoli* isolate JP35B-1X (MG649292.1) | 99.83 | *Valsaaceae* |
| B24 | PQ614818 | *Nigrospora oryzae* strain HBM1 (JN211105.1) | 99.82 | *Apiosporaceae* |
| A92 | PQ614819 | *Botryosphaeria ramosa* CMW 26167 (NR_151841.1) | 98.62 | *Botryosphaeriaceae* |
| A85 | PQ614820 | *Neofusicoccum parvum* isolate CO2-2 (MH265978.1) | 100.00 | *Botryosphaeriaceae* |
| P9 | PQ614821 | *Pestalotiopsis mangiferae isolate GXIMD 03165* (OK036728.1) | 100.00 | *Sporocadaceae* |

Table S2 List of PCR primers used for viral contig validation

| Contig number | Virus | Primer name | Primer reverse (5'-3') | Product size (bp) |
| --- | --- | --- | --- | --- |
| Contig36167 （ORF1） | Neofusicoccum parvum narnavirus 4 (RNA1) | NarF1768 | GTGGTGAAGGAGCCGGGTAA | 587 |
|  |  | NarR2354 | AGCCTCCTTCCTACGTGTCGTG |  |
| Contig36167 （ORF2） | Neofusicoccum parvum narnavirus 4 (RNA2) | NarF2598 | CGGTTCACTGAGACACCACTCAC | 505 |
|  |  | 36167A | TCGTCGCCTTCCCAACC |  |
| Contig36685 | Neofusicoccum parvum narnavirus 4 (RNA3) | 36685B | CGTAGCATCACTTTTCACCACC | 494 |
|  |  | 36685A | TGAGCCCTGGTCATACCCTC |  |
| Contig39786 | Pestalotiopsis mangiferae deltaflexivirus 1-P9 | 39786B | ACTGGGAGTGACCTGATGAATG | 566 |
|  |  | 39786A | TCCAAATACCCGTCGTGAGAT |  |
| Contig39771 | Phomopsis phaseoli alternavirus 1 (dsRNA1) | 39771B | CATTATCACGACATCTGCTCCTT | 707 |
|  |  | 39771A | GTGCCCCACTTATGTTTCCA |  |
| Contig39474 | Phomopsis phaseoli alternavirus 1 (dsRNA2) | 39474B | CGGTTTTGGTATCATTTTGTCG | 716 |
|  |  | 39474A | AGTACCCGCAGATTCCGTTCA |  |
| Contig39168 | Phomopsis phaseoli alternavirus 1 (dsRNA3) | 39168B | GGAAGTCTTGGCGATGTTGC | 586 |
|  |  | 39168A | AGTCATGCTGTGCGGGATGA |  |
| Contig38550 | Phomopsis phaseoli alternavirus 1 (dsRNA4) | 38550B | TTCCGCCCGATAAAGCAG | 647 |
|  |  | 38550A | GCGAACATCCGAACCAGC |  |
| Contig34766 | Pestalotiopsis mangiferae chrysovirus 1 (dsRNA1) | 34766B | GGACCAGTTGGTCGGTAGGAG | 739 |
|  |  | 34766A | GAACATGGCGTAATCTGTCGG |  |
| Contig39301 | Pestalotiopsis mangiferae chrysovirus 1 (dsRNA2) | 38856B | AGAATCGCTGTGCCAGACG | 544 |
|  |  | 38856A | CGCACTATCTACCACCACTTCC |  |
| Contig38856 | Pestalotiopsis mangiferae chrysovirus 1 (dsRNA3) | 39301B | AGTAAGAGGGTCCACCACAATG | 719 |
|  |  | 39301A | CTGCCACCGAGAAAACACC |  |
| Contig39201 | Pestalotiopsis mangiferae chrysovirus 1 (dsRNA4) | 39201B | CCTACGATGACTAGACGTGCTGT | 677 |
|  |  | 39201A | GAGGTTATCGGCACATACTCATC |  |
| Contig37831 | Neofusicoccum parvum chrysovirus 2 (dsRNA1) | 37831B | GGCGAAGCAATCCAAACC | 843 |
|  |  | 37831A | GTGAAAACCCACAAGCATCTCC |  |
| Contig36161_g1 | Neofusicoccum parvum chrysovirus 2 (dsRNA2) | 36161B | AGAGCAATCATTTCCTTCGGT | 613 |
|  |  | 36161A | TATACCAGGTAACCATCTCGTCAG |  |
| Contig32303 | Neofusicoccum parvum chrysovirus 2 (dsRNA3) | 32303B | TCTGCACGTCGGGACTGA | 716 |
|  |  | 32303A | TGCTCGTTGCGGTGGTC |  |
| Contig36161_g2 | Neofusicoccum parvum chrysovirus 2 (dsRNA4) | 36161D | GGCGTCGGGGACTGTTTA | 685 |
|  |  | 36161C | CGGGCGTATTGATTGACTGG |  |
| Contig34008 | Phomopsis partitivirus 3 (dsRNA1) | 34008B | CTGTTATGGATTGGGAATGGAA | 532 |
|  |  | 34008A | GAGAATCGTTTTGCTGGTGC |  |
| Contig29582 | Phomopsis partitivirus 3 (dsRNA2) | 29582B | GTAGCCGACGCTAAACCCTAC | 518 |
|  |  | 29582A | GGTGCCGCCGATGAGA |  |
| Contig8561  (92contig9071) | Botryosphaeria ramosa polymycovirus 1 (dsRNA1) | 8561B | GGTTACGTCGCGTCGTGATC | 930 |
|  |  | R1767 | CGCGAGCAGAACCTGACGC |  |
| Contig4706  (92contig9284) | Botryosphaeria ramosa polymycovirus 1 (dsRNA2) | 4706B | TCAGCGTGTCCGCACCA | 420 |
|  |  | 4706A | CGAGCACAAGATCGCATAGAC |  |
| Contig27821  (92contig9395) | Botryosphaeria ramosa polymycovirus 1 (dsRNA3) | 27821B | CGTGTACGAGATGGGGTCAG | 670 |
|  |  | 27821A | ATCAGGGTCGCCAAGCAT |  |
| 92contig7847 | Botryosphaeria ramosa polymycovirus 1 (dsRNA4) | hpF | CCCTGGATGTTCTCACCGGT | 296 |
|  |  | hpR | GCCAAGCGGTCTTACACAGG |  |
| Contig16704 | Nigrospora oryzae Victoriavirus 2-B24 (ORF1) | 36111B | CCCGAAACCGACGAGACAG | 627 |
|  |  | 36111A | CGGAGCCACAGAGGGATAGC |  |
| Contig36111 | Nigrospora oryzae Victoriavirus 2-B24 (ORF2) | 16704B | CGCTTTCCGCAGTCTAATCTC | 553 |
|  |  | 16704A | CGGGTTCACATGGTTGGC |  |
| Contig38185 | Pestalotiopsis mangiferae bunyavirus 1 | 38185A | GTCAGTCTACCAGCCACCAAAG | 611 |
|  |  | 38185B | TGCCTGACAGGTGATAAATCGA |  |
| Contig38664 | Nigrospora oryzae discovirus 1 (RNA1) | 38664B | GCTCTTTCGAGGCCCCAC | 565 |
|  |  | 38664A | ACGACTTCGGCGTCTACCC |  |
| Contig38293 | Nigrospora oryzae discovirus 1 (RNA2) | 38293B | TGAGTTGGTTTTCAGGGTTGTG | 501 |
|  |  | 38293A | AGATTACGGAGCAGAGTCGGA |  |
| Contig37620 | Nigrospora oryzae discovirus 1 (RNA3) | 36720A | GCAGAAGACGCTGACGGAG | 511 |
|  |  | 36720B | AGTGACGAGCCCTACAAGAAAG |  |

Table S3 List of primers used for viral terminal amplification

| Targeted virus1 | Primer name | Sequence (5'-3') | length (bp)^1^ | Purpose |
| --- | --- | --- | --- | --- |
| Neofusicoccum parvum narnavirus 4 (RNA1) | NarR182 | CCTGCTTCGTCGAAAGGTAGTG | 207 | 5' end |
|  | NarF2197 | TCTACGCGACTGGCGTATTG | 327 | 3' end |
| Neofusicoccum parvum narnavirus 4 (RNA2) | 36167A | TCGTCGCCTTCCCAACC | 566 | 5' end |
|  | NarF3567 | CGAGCGTGAGTGGGAGAATAC | 1212 | 3' end |
| Neofusicoccum parvum narnavirus 4 (RNA3) | Na2R262 | CCCGCAATCACTACTACGAAAGA | 196 | 5' end |
|  | Na2F698 | CACGAAGTCTGGACCGTTGTC | 252 | 3' end |
| Pestalotiopsis mangiferae deltaflexivirus 1-P9 | DVR149 | CATTCCCAGGCTGTCCAAC | 149 | 5' end |
|  | DVF7396 | TTCCAACTCTTTCACTGATAGGC | 345 | 3' end |
| Phomopsis phaseoli alternavirus 1 (dsRNA1) | AV1R178 | GCGATTGTCGTTTGAGTGCA | 186 | 5' end |
|  | AV1F3442 | TTGTTTGGACTTCTGCTTCAGG | 232 | 3' end |
| Phomopsis phaseoli alternavirus 1 (dsRN2) | AV2R188 | GAAATGCAGGCAAGCCACA | 196 | 5' end |
|  | AV2F2490 | TGGTATTGGAGGTGGAAGGAG | 181* | 3' end |
| Phomopsis phaseoli alternavirus 1 (dsRNA3) | AV3R316 | GGTCCTCCTTTGCTACCTCTG | 271 | 5' end |
|  | AV3F277 | CGCTTGGTGGCAGGTGTC | 241 | 3' end |
| Phomopsis phaseoli alternavirus 1 (dsRNA4) | AV4R295 | GCGAGTGTCCAGGTGCTGA | 289 | 5' end |
|  | AV4F1616 | AGGGCACCCAGACTTTTCGC | 90 | 3' end |
| Pestalotiopsis mangiferae chrysovirus 1 (dsRNA1) | PCh1R2 | CGAACTCAGCCTTAATCGCATC | 248 | 5' end |
|  | PCh1F3 | TGCTATGTTACATGGCATCGAG | 118 | 3' end |
| Pestalotiopsis mangiferae chrysovirus 1 (dsRNA2) | PCh3R1 | CCCTCAAGTCGCACTAGAACAG | 153 | 5' end |
|  | PCh3F6 | AAACGCTGTCAAATTACGCTAGG | 115 | 3' end |
| Pestalotiopsis mangiferae chrysovirus 1 (dsRNA3) | PCh2R1 | CACTGGTTCCTTCGCCGTAC | 136 | 5' end |
|  | PCh2F2 | AGAGGCTCAAGAGGTATGCTGTA | 74 | 3' end |
| Pestalotiopsis mangiferae chrysovirus 1 (dsRNA4) | PCh4R5 | ATGCACTACCACCAGTCCCTTC | 465 | 5' end |
|  | PCh4F4 | CTCAGGAGCAAAATGCCAGTA | 769 | 3' end |
| Neofusicoccum parvum chrysovirus 2 (dsRNA1) | C1R281 | CGAAGCACCGAGTGTAGCAGAC | 238 | 5' end |
|  | C1F3361 | AAGCACCCTTACCGGAAGAT | 330 | 3' end |
| Neofusicoccum parvum chrysovirus 2 (dsRNA2) | C2R182 | CGTCGTTAATCACGGGTTGT | 176 | 5' end |
|  | C2F2396 | GAACAGCAGGCTTACGACAAC | 331 | 3' end |
| Neofusicoccum parvum chrysovirus 2 (dsRNA3) | C3R294 | TAGGAGCACATGGTGTTGGGA | 294 | 5' end |
|  | C3F2386 | GCCCACAGATCACAACCCTG | 207 | 3' end |
| Neofusicoccum parvum chrysovirus 2 (dsRNA4) | C4R149 | GCTTGTGAGCGTGCGGAGAG | 149 | 5' end |
|  | C4F2174 | GTCTGACCCCGGTCTACAACA | 419 | 3' end |
| Phomopsis partitivirus 3 (dsRNA1) | PVRR281 | GCCATCAAGGCATCTTTTCG | 178 | 5' end |
|  | PVRF1512 | CTTCAAATACCTTGTCACCCTG | 324 | 3' end |
| Phomopsis partitivirus 3 (dsRNA1) | PVCR119 | CATGACTGTCGATGATGGTGC | 121 | 5' end |
|  | PVCF1438 | ACGACCCTTGGCTCTTCACG | 306 | 3' end |
| Botryosphaeria ramosa polymycovirus 1 (dsRNA 1) | PM1R133 | CGAGCGAGAAGGAATCAGACAT | 140 | 5' end |
|  | PM1F2050 | CCGACCAGTCCATTGCGAT | 368 | 3' end |
| Botryosphaeria ramosa polymycovirus 1 (dsRNA 2) | PM2R400 | CGAGAACAACGACTCAACCAC | 418 | 5' end |
|  | PM2F1500 | CCCAAGTTGACTTCGGCTCC | 285 | 3' end |
| Botryosphaeria ramosa polymycovirus 1 (dsRNA 3) | PM3R209 | GGTGACTCCGCACGCTCCTC | 326 | 5' end |
|  | PM3F1763 | TGGTGATCTGTCTGGTGGTGTG | 307 | 3' end |
| Botryosphaeria ramosa polymycovirus 1 (dsRNA 4) | PM4F1338 | GGTGGGGAGTTGGCGAGATA | 93 | 5' end |
|  | PM4R185 | GTTGTGGGGGTATAAGCGGACG | 252 | 3' end |
| Nigrospora oryzae Victoriavirus 2-B24 | vicR125 | GACGGACTAGATCGCTCTTCG | 216 | 5' end |
|  | vicF4561 | GTTGAGGCAGCCGCAATTG | 506 | 3' end |
| Pestalotiopsis mangiferae bunyavirus 1 | PNVF6884 | CTCATCTGGGACAAGTCTCAATCT | 298 | 5' end |
|  | PNVR261 | AACAATCGCAAGCAGGAAGTAG | 270 | 3' end |
| Nigrospora oryzae discovirus 1 (RNA1) | B1LF6265 | TGCCCATTGAGCTTCTTGTT | 358 | 5' end |
|  | B1LR382 | AAGAGCGGAAAATTCTACCACA | 364 | 3' end |
| Nigrospora oryzae discovirus 1 (RNA2) | B1NF1089 | ATGTTGATGGGGCTTAACCTAA | 188 | 5' end |
|  | B1NR383 | CAAGACTCAGCGCCTCCAAC | 353 | 3' end |
| Nigrospora oryzae discovirus 1 (RNA3) | B2NF1948 | ATGCCACCGCTGCCATAC | 559 | 5' end |
|  | 38293A | AGATTACGGAGCAGAGTCGGA | 999 | 3' end |
|  | PC3T7loop | GGATCCCGGGAATTCGGTAATACGACTCACTATATTTTTATAGTGAGTCGTATTA |  | Universal primer |
|  | PC2 | CCGAATTCCCGGGATCC |  | Universal primer |

^1^ Virus sequence length without the termini polynucleotide, such as polyadenine or polyuracil.

Table S4 List of primers used for contigs gap amplification

| Primer name | Sequence (5'-3') | Size (bp) | Target gap |
| --- | --- | --- | --- |
| vicF2866 | GATTCGTCGTTCAGAAACAAGC | 1266 | Nigrospora victorivirus (between contig36111 and contig16704) |
| vicR4131 | TGTAGGGTTCATGCGACAGC |  |  |

Table S5 List of accession numbers and full virus names used for sequence alignments

| Virus name | Virus abbrev | GenBank accession number |
| --- | --- | --- |
| Neofusicoccum parvum narnavirus 4-RNA1 | NpNarV4-RNA1 | PQ653950 |
| Neofusicoccum parvum narnavirus 4-RNA2 | NpNarV4-RNA2 | PQ653952 |
| Botrytis cinerea binarnavirus 2-RNA1 | BcBNV2-RNA1 | QJT73725.1 |
| Botrytis cinerea binarnavirus 2-RNA2 | BcBNV2-RNA2 | QLF49184.1 |
| Downy mildew lesion associated splipalmivirus 3 RNA1 | DmLASV3-RNA1 | QIR30282.1 |
| Downy mildew lesion associated splipalmivirus 3 RNA2 | DmLASV3-RNA2 | WNA22209.1 |
| Aspergillus fumigatus narnavirus 1 | AfuNV1 | AXE72933.1 |
| Zhangzhou Narna tick virus 3-RNA1 | ZzNTV | UYL95381.1 |
| Neofusicoccum parvum narnavirus 2 | NpNV2 | QDB74995.1 |
| Neofusicoccum parvum narnavirus 3 | NpNV3 | QTE76053.1 |
| Saccharomyces 23S RNA narnavirus | ScNV-23S | NP 660177.1 |
| Saccharomyces 20S RNA narnavirus | ScNV-20S | NP 660178.1 |
